# The relationship between real-time ultrasound-detected extra-articular soft tissue pathologies and knee pain in osteoarthritis: a cross-sectional study

**DOI:** 10.1007/s10067-026-08017-x

**Published:** 2026-03-06

**Authors:** Asim Ghouri, Luke Budworth, Richard J. Wakefield, Sarah R. Kingsbury, Philip G. Conaghan

**Affiliations:** 1 Leeds Institute of Rheumatic and Musculoskeletal Medicine, University of Leeds, Leeds, UK; 2 NIHR Leeds Biomedical Research Centre, Leeds Teaching Hospitals NHS Trust, Leeds, UK; 3 Bradford Institute for Health Research, Bradford, UK

**Keywords:** Knee, Ligament, Osteoarthritis, Pain, Tendon, Ultrasound

## Abstract

**Objective:**

Extra-articular soft tissue pathologies are relatively understudied in osteoarthritis (OA). We aimed to explore the relationship between structural pathology, sites of knee pain and knee pain severity, as determined using real-time ultrasound (US).

**Methods:**

Patients with painful knee OA received an US scan assessing a range of soft tissue structures at four quadrants (anterior, medial, lateral and posterior). US probe site tenderness (sono-palpation) was also assessed at each structure. Participants’ reported outcome measures were recorded, including sites of knee pain using the Knee Pain Map (using the same quadrant locations as structural pathology) and average numeric rating scale (NRS) for pain severity in the past week.

**Results:**

Fifty-four knees (26 men, BMI 31.5, mean NRS pain 5.4, 96% Kellgren–Lawrence 2/3) were included. Medial pain was the most frequently reported location. Semimembranosus tendon (SMT) hypoechogenicity, indicating tendinopathy, was the most frequent US finding (53%). However, there was no clear pattern between SMT pathology and knee pain location. Medial collateral ligament (MCL) and lateral collateral ligament (LCL) sono-palpation (reflecting clinical enthesitis) was more frequently observed in medial and lateral knee pain, respectively (43.2% and 52.9%). Higher NRS pain was significantly associated with MCL sono-palpation (1.41 NRS points; CI 0.47–2.36, *p* = 0.004) and Baker’s cyst sono-palpation (1.78 NRS points; CI 0.35–3.20, *p* = 0.016). A trend toward greater NRS pain was associated with US-detected patellar tendon pathology (1.1 NRS points; CI − 0.14–2.35, *p* = 0.08).

**Conclusion:**

This exploratory study highlighted the potential importance of extra-articular knee pathologies, particularly the collateral ligaments, in understanding OA knee pain location and severity.

**Table:** 

**Key Points** • *This study measured the potential role of extra-articular pathology in OA knee pain.* • *Sono-palpation has been used as a novel method of measuring enthesitis.* • *Medial and lateral collateral ligament enthesitis may contribute to respective joint pain locations in knee OA.* • *Enthesitis may be secondary to biomechanical stresses around the knee joint.*

**Supplementary Information:**

The online version contains supplementary material available at 10.1007/s10067-026-08017-x.

## Introduction

Pain is the major symptom experienced by people with knee osteoarthritis (OA); it is complex and multifactorial [1]. It remains unclear which tissue structures contribute to pain, and pain correlates only weakly with radiographic OA. MRI has identified several pain-related structures, mainly intra-articular features [2–4], while musculoskeletal ultrasound (US) provides a non-invasive, real-time assessment of periarticular tissues, though evidence linking US-detected pathology to OA knee pain remains limited and focused largely on global pain and intra-articular changes [5–9].

We hypothesised that extra-articular soft tissue pathology contributes to OA knee pain and that pain location may indicate its presence. This study therefore explored associations between US-detected extra-articular pathologies and both severity and site of pain in people with moderately to severely painful knee OA.

## Patients and methods

Ethical approval was obtained from Black Country Research Ethics Committee (IRAS ID 260194, REC number 19/NW/0205), and all patients provided written informed consent for study participation.

Participants from primary and secondary care were included between February 2021 and May 2022 if aged 50 and over, with any radiographic evidence (anterior–posterior and lateral X-ray views) of knee OA (Kellgren–Lawrence (KL) grades 1–4) and a visual analogue scale (VAS) pain severity in the past 3 months of ≥ 40/100. Exclusions included having surgery or knee injury in the past 6 months, inflammatory arthritis, previous partial or total knee replacement or recent intra-articular, intramuscular or oral steroids.

### Clinical evaluation

Demographic parameters recorded included age, sex, BMI and ethnicity. Participants completed a numeric rating scale (0–10 NRS) for average knee pain over the past week and the Knee Pain Map (KPM) [10]. Higher NRS values indicate greater pain severity (0 = no pain, 10 = worst pain). Knee pain location was categorised as medial, lateral, patellar, or back of the knee or diffuse (Supplementary Fig. 1). Pain duration and presence of quadriceps weakness on clinical examination (derived from Medical Research Council grading) were also recorded.

### Sonographic evaluation

Grey scale and Doppler ultrasound were performed using an Aixplorer (Supersonic Imagine, Aix-en-Provence, France) system, operated by a single trained ultrasonographer with over 25 years musculoskeletal ultrasound experience, using a standardised SL18-5 MHz probe (5-cm length, 1-cm width). To reflect KPM regions, extra-articular structures were evaluated using anterior, medial, lateral and posterior knee quadrants (Supplementary Table 1 and Supplementary Fig. 2). These included the quadriceps tendon, patellar tendon and superficial and deep infrapatellar bursa (anterior); medial collateral ligament (MCL) and pes anserine bursa (medial); lateral collateral ligament (LCL) and iliotibial band (ITB, lateral); and semimembranosus tendon (SMT) and Baker’s cyst (posterior) (Supplementary Fig. 2). Probe positioning is demonstrated in Supplementary Fig. 3.

**Fig. 1:**
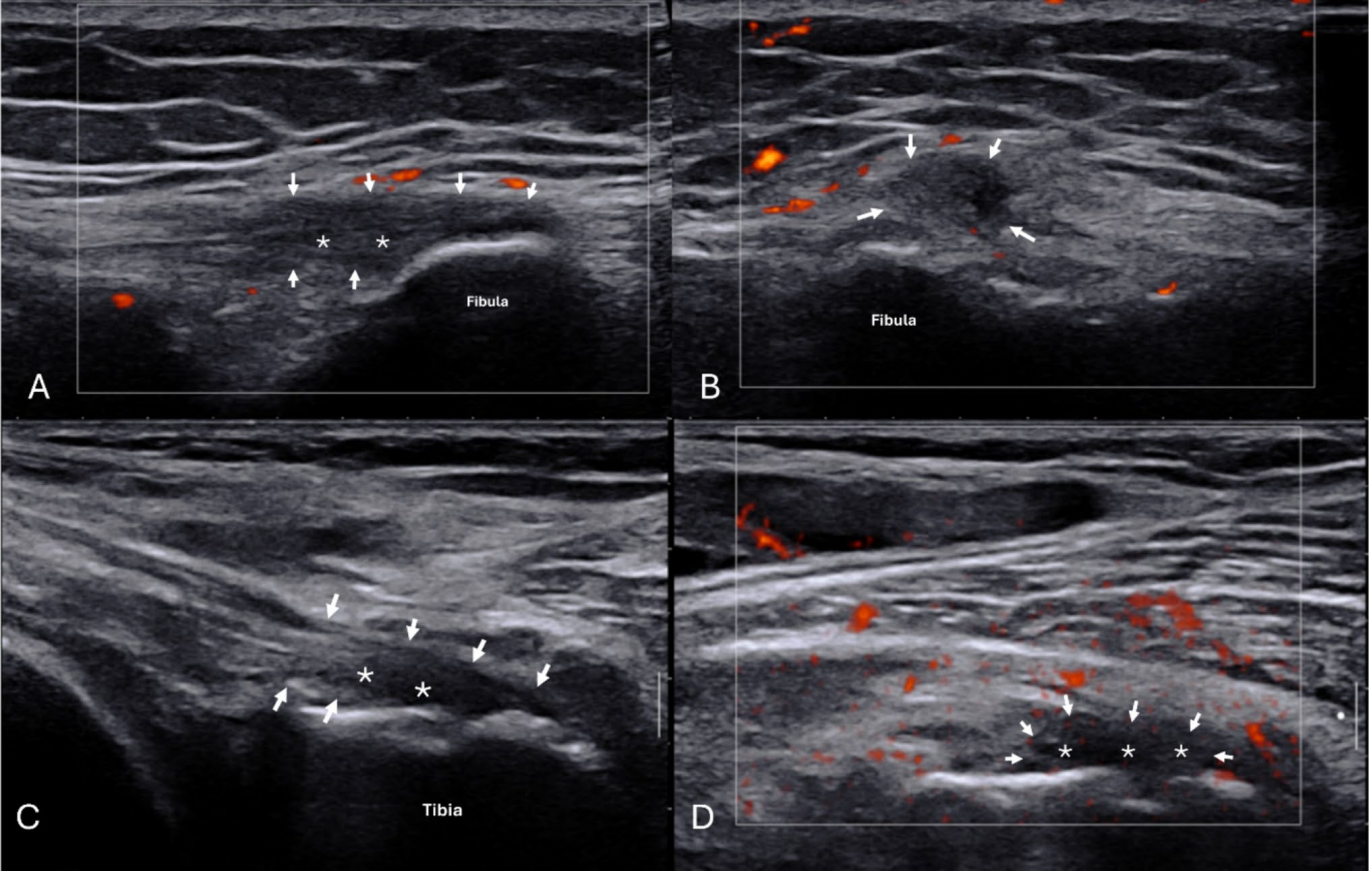
LCL hypoechogenicity and SMT hypoechogenicity. Real-time ultrasound imaging of ligament and tendon hypoechogenicity. **A**, **B** Lateral collateral ligament (LCL) hypoechogenicity at insertional fibula (white arrows), with incidental power Doppler signal in surrounding soft tissues. **C**, **D** Semimembranosus tendon (SMT) insertion hypoechogenicity (asterisks). Hypoechogenicity indicates structural enthesopathy and may contribute to localised knee pain

Ultrasound characteristics assessed included presence/absence of specific pathologies like tendon tears and structural changes related to enthesitis as per Outcome Measures in Rheumatology (OMERACT) definitions [11, 12]: thickening/enlargement, hypoechogenicity (darker grey on US), power Doppler (PD) grade of the structure, insertional erosions and enthesophytes. Some US characteristics (grade and PD grade) were scored 0–3 to reflect their severity (subsequently simplified to a binary variable for analysis) while the remaining characteristics were scored 0 or 1 to demonstrate their absence or presence (Supplementary Table 1). The binary scoring of the enthesitis variables was in keeping with OMERACT scoring [11]. Figure 1 shows LCL and SMT hypoechogenicity. Supplementary Fig. 4 shows an enthesophyte. In addition to entheseal-related structures, Baker’s cysts and bursae were also assessed. The US probe was also used to detect probe site tenderness (sono-palpation) over tendons, ligaments, Baker’s cysts and bursae. This was applied with a similar level of pressure typically used when palpating joint tenderness on clinical examination. Sono-palpation is a well-described dynamic method of integrating palpation with real-time imaging [13].

### Statistical analysis

In this exploratory study, statistical analyses were performed using R Studio (Posit, Boston, MA). Baseline characteristics were described using descriptive statistics. For analysis, localised and regional pain were grouped together into corresponding quadrants (anterior, medial, lateral and posterior) defined as ‘mapped regions’. NRS pain was cross-tabulated with KPM frequencies to explore associations. We conservatively used a binary presence/absence of pathology variable instead of a severity score. This binary approach simplifies models and reduces dimensionality compared to using severity scores, potentially reducing power to detect dose-dependent effects but improving interpretability.

To determine whether there was a relationship between the US-identified pathologies and pain, we performed multivariable linear regression models, where each pathology was the main predictor (pathology present or absent) and age, sex and BMI were control variables. Greater emphasis was placed on minimising type II error (false negatives), with an increased type I error risk (false positives) accepted as a methodological compromise, and multiplicity was therefore not adjusted for. The large number of tests performed in this exploratory study was undertaken to generate preliminary signals for future hypothesis generation. The origins and insertions of some ligaments and tendons were combined in analyses to reflect pathology of a single structure and to ensure an adequate sample size.

## Results

Fifty-four participants (26 male, 28 female) were enrolled. Mean age was 64.07 (SD 8.03) and mean BMI 31.45 (SD 5.42). Fifty-one participants were white, two Black-Caribbean and one Chinese. Two participants were current smokers, 22 ex-smokers and 30 never smoked. 51/54 (94%) participants had KL grade 2 or 3. Medial compartment OA was present in 53/54 participants, lateral knee OA in 23 participants and patellofemoral OA in 43 participants. Mean NRS pain over the past week was 5.41 (SD 1.66).

Thirty-six participants reported medial pain, 16 participants reported lateral pain, 10 participants reported anterior knee pain, 5 participants reported posterior pain and 5 participants reported diffuse pain. Participants could have > 1 area of pain, but 27 participants (50%) had pain in only one region.

Supplementary Table 2 shows the frequency of each US pathology and the frequency of sono-palpation (sono-palpation frequency per knee regions demonstrated in Supplementary Fig. 5). SMT hypoechogenicity was the most frequent finding (53% of knees scanned), with SMT insertion the most frequent tender point (66% knees). Some US structural pathology measurements were missing due to user input error during data entry, as opposed to difficulties with image acquisition or interpretation. Missing data was reported for one participant for the following pathologies: medial collateral ligament PD grade; Baker’s cyst sono-palpation; SMT thickening/enlargement, hypoechogenicity, PD grade and insertional erosions. SMT enthesophyte data was missing for two participants and SMT tear data was missing for four participants. Missing data for the relevant structure resulted in a reduced denominator when calculating the frequency of pain for that region.

Table 1 shows the pathologies with the highest frequency for each pain region (localised and regional pain combined). The full dataset is in Supplementary Table 3. SMT insertion hypoechogenicity was the pathology with the highest proportion of presence within a pain region (82.4% of participants with lateral regional pain).

**Table 1:** Frequency of pathology for each knee pain region^*^

Pain region	Pathology	Total frequency^†^	Pain region frequency^‡^
Anterior	Semimembranosus tendon hypoechogenicity	53.7% (29/53)	55.6% (5/9)
	Medial collateral ligament sono-palpation	38.9% (21/54)	50% (5/10)
	Quadriceps tendon enthesophytes	51.9% (28/54)	50% (5/10)
	Semimembranosus tendon sono-palpation	66.7% (36/54)	50% (5/10)
	Semimembranosus tendon enthesophytes	33.3% (18/52)	44.4% (4/9)
Medial	Semimembranosus tendon sono-palpation	66.7% (36/54)	70.3% (26/37)
	Quadriceps tendon enthesophytes	51.9% (28/54)	56.8% (21/37)
	Semimembranosus tendon hypoechogenicity	53.7% (29/53)	54.1% (20/37)
	Medial collateral ligament sono-palpation	38.9% (21/54)	43.2% (16/37)
	Semimembranosus tendon enthesophytes	33.3% (18/52)	41.7% (15/36)
Lateral	Semimembranosus tendon sono-palpation	66.7% (36/54)	82.4% (14/17)
	Semimembranosus tendon hypoechogenicity	53.7% (29/53)	81.2% (13/16)
	Baker’s cyst thickening	46.3% (25/54)	52.9% (9/17)
	Lateral collateral ligament sono-palpation	37.0% (20/54)	52.9% (9/17)
	Semimembranosus tendon thickening	31.5% (17/53)	50% (8/16)
Posterior	Baker’s cyst thickening	46.3% (25/54)	80% (4/5)
	Semimembranosus tendon sono-palpation	66.7% (36/54)	80% (4/5)
	Quadriceps tendon enthesophytes	51.9% (28/54)	60% (3/5)
	Baker’s cyst presence	37.0% (20/54)	60% (3/5)
	Medial collateral ligament hypoechogenicity	24.1% (13/54)	40% (2/5)
	Semimembranosus tendon enthesophytes	33.3% (18/52)	40% (2/5)
	Semimembranosus tendon thickening	31.5% (17/53)	40% (2/5)
	Semimembranosus tendon hypoechogenicity	53.7% (29/53)	40% (2/5)
Diffuse	Quadriceps tendon enthesophytes	51.9% (28/54)	60% (3/5)
	Medial collateral ligament enthesophytes	20.4% (11/54)	60% (3/5)
	Baker’s cyst thickening	46.3% (25/54)	60% (3/5)
	Baker’s cyst presence	37.0% (20/54)	40% (2/5)
	Lateral collateral ligament hypoechogenicity	38.9% (21/54)	40% (2/5)
	Lateral collateral ligament thickening	33.3% (18/54)	40% (2/5)
	Medial collateral ligament hypoechogenicity	24.1% (13/54)	40% (2/5)
	Medial collateral ligament thickening	18.5% (10/54)	40% (2/5)
	Semimembranosus tendon hypoechogenicity	53.7% (29/53)	40% (2/5)
	Semimembranosus tendon sono-palpation	66.7% (36/54)	40% (2/5)
	Semimembranosus tendon thickening	31.5% (17/53)	40% (2/5)

^*^Only top five pathology frequencies are shown for each pain region

^†^Out of total number of participants in the study with available data

^‡^Percentages for pain region frequency are calculated as the number of participants with the specified structural pathology (numerator) out of the total number of participants reporting pain in that region or diffuse pain with non-missing data for that pathology (denominator). Denominators vary between pathologies within a pain region because frequencies are based on available cases for each ultrasound variable

**Fig. 2:**
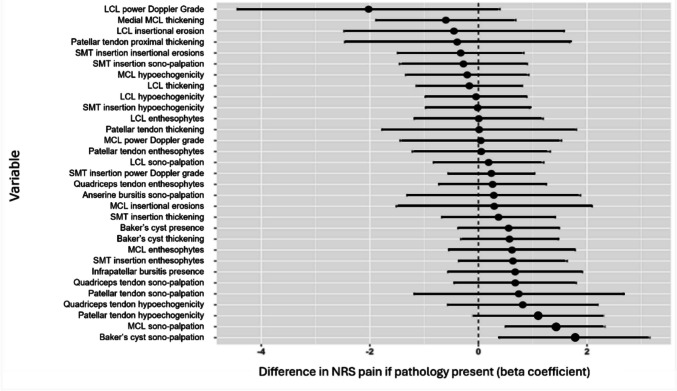
Associations of structural pathologies and average global knee NRS pain^*^ (linear regression models, adjusted for age, sex and BMI). ^*^Variables with < 5% prevalence were excluded

Figure 2 shows the association of US findings with degree of NRS pain. While no visualised pathologies were significant after adjustment, greater NRS pain was observed with patellar tendon hypoechogenicity (1.1 NRS points; CI − 0.14–2.35, p = 0.08). Participants with MCL (1.41 NRS points; CI 0.47–2.36, *p* = 0.004) and Baker’s cyst (1.78 NRS points; CI 0.35–3.20, *p* = 0.016) sono-palpation were significantly more likely to have greater global knee pain. Knee pain chronicity was not found to have an effect on NRS pain. Forty-eight participants had quadriceps strength data recorded; weakness was present in 36 (75%) and absent in 12 (25%). There was no significant difference in NRS pain between groups, although the high prevalence of quadriceps weakness limited meaningful analysis.

## Discussion

This is the first US study to focus on multiple extra-articular soft tissue structures and their association with OA knee pain. This is also the first study to assess sono-palpation (similar to examination for clinical enthesitis but with more precision) of knee structures in OA.

In measuring the relationship between specific pathology and pain regions, the highest proportion of pathology was found with SMT hypoechogenicity in the presence of pain in the lateral region. Hypoechogenicity is consistent with structural enthesopathy [12]. More severe degenerative changes in the SMT have also been demonstrated by histological and ultrastructural analysis in moderate-to-severe knee OA compared to patients with mild-to-moderate knee OA [14].

Although posterior knee pain was less commonly reported, a high proportion of these patients had Baker’s cysts. While Baker’s cysts have not previously been linked to global knee pain [15], our finding relates to a specific, less prevalent posterior pain region and may reflect direct pressure from the cyst. Cyst thickening may also indicate more widespread effusion or synovitis [16].

Medial knee pain was the most common site of pain, consistent with the predominant medial compartment involvement in this study and in keeping with previous literature [17, 18]. SMT insertion hypoechogenicity was the most frequent US-detected pathology, although it was not clearly related to a specific compartment. Given its high frequency, there is a possibility that SMT hypoechogenicity may reflect alterations in gait due to OA knee pain and causing biomechanical overload and increased tensile forces. A higher prevalence of semimembranosus tendinopathy has been found in a previous MRI study of medial knee pain (compared to controls without pain) though the OA status of subjects was not reported [19]. However, in that report, a higher prevalence of pes anserine bursitis was also found which was not detected in our study. When analysing medial and lateral regions, which included more participants than other pain regions, tenderness of the corresponding collateral ligaments was more frequent.

Our data has shown the potential importance of extra-articular structures in contributing to OA knee pain. A wide variety of soft tissue pathologies were evaluated in this study, but our data suggests the patellar tendon was potentially associated with greater global OA knee pain. This may reflect increased biomechanical forces on this structure in knee OA secondary to quadriceps weakness, a frequent finding in knee OA [20]. A recent study by Ziegeler et al. using PET-MRI has also demonstrated an association between increased patellar tendon enthesis uptake and greater KOOS pain in patellofemoral OA [21]. Our study also shows a relatively high frequency of enthesophytes at the quadriceps tendon insertion, which may be related to repetitive strain and bone pulling [22, 23]. Knee enthesophytes have not been widely studied in knee OA, particularly with respect to the quadriceps tendon, though enthesopathy-related OA has previously been suggested as a specific OA phenotype [24, 25]. Deep learning models of a large MRI dataset have identified the medial patellar retinaculum, gastrocnemius tendon and plantaris muscle as potential structures associated with increased risk of total knee replacement over 5 years, further highlighting the importance of extra-articular tissues in symptomatic knee OA [26].

Increased sono-palpation frequency was observed over structures including the SMT, MCL and LCL. These structures were also present in higher proportions in the quadrants where patients reported pain and therefore may be relevant to a patient’s daily knee pain. This further highlights the potential role of enthesitis in contributing to knee pain. This may also have implications on future OA knee pain treatment strategies with increased focus on mechanical unloading of these structures.

This study had several novel aspects, including differentiating pain via anatomical locations and assessing extra-articular structures including the quadriceps tendon, patellar tendon, collateral ligaments and ITB. The use of sono-palpation ensured tenderness was specifically associated with the intended assessed structure, providing greater reliability compared to clinical palpation. Tenderness may also detect pathology that is too microscopic to be detected on imaging. Sono-palpation has been investigated previously for assessing lateral epicondylitis in the elbow [27]. MSK US is also a widely adopted and versatile tool in clinical practice, with applications extending beyond diagnosis, such as the characterisation of inflammation within the knee, which can directly inform therapeutic decision-making [28].

This study has several limitations. It was an exploratory, cross-sectional study and therefore cannot establish a causal relationship between structural pathology and pain. Findings may have been influenced by confounding factors affecting pain levels at the time of assessment (e.g. recent physical activity). The sample size was small, reflecting the study’s exploratory nature. All ultrasound examinations were performed by a single, highly experienced ultrasonographer from the EULAR OMERACT Ultrasound Working Group, but intra-rater reliability was not assessed. When assessing sono-palpation, the US probe may have compressed adjacent structures. Excessive transducer pressure could also have led to over-detection of tenderness; however, using the same method in healthy volunteers (data not shown) produced tenderness in only one of 30 individuals. Although care was taken to keep the transducer perpendicular to the structure to minimise artefact, some findings may still reflect anisotropy. This may explain the high frequency of SMT hypoechogenicity, as it attaches to bone at an angle and is therefore less perpendicular to the US probe compared to the collateral ligaments.

Earlier US studies examining the relationship between structural pathology and pain identified intra-articular findings, such as synovial pathology and meniscal extrusion, which were not evaluated in the current study [6]. US may also have utility when assessing the cruciate ligaments [29]. The lack of strong structure–pain associations may of course reflect unmeasured intra-articular features (e.g. synovitis) given our focus on extra-articular tissues. US also has limited ability to visualise deeper structures, restricting the range of assessable features. Pain is complex and influenced by psychosocial factors and peripheral/central sensitisation. Including only participants with painful OA may introduce collider bias (where analyses are restricted to individuals with a particular outcome, leading to spurious or exaggerated associations between its causes). This may therefore have potentially overestimated associations between structural pathology and knee pain, and selecting those with VAS ≥ 40/100 may have further exacerbated this. The large number of variables and relatively small sample may have reduced power to detect true associations, while multiple testing could yield false positives. It is legitimate not to adjust for multiple comparisons in exploratory studies, given this study is explicitly framed as hypothesis-generating rather than confirmatory. Findings should therefore be considered exploratory, highlighting structures of interest for future research. Future work should also assess intra-articular US features and examine whether gait and knee alignment mediate structure–pain relationships in OA. Future studies should also include participants across a broader pain spectrum, including those with structural OA but minimal pain, to reduce the likelihood of collider bias.

This exploratory study has highlighted the potential importance of extra-articular knee pathologies, particularly the collateral ligaments, in understanding OA knee pain location and severity.

## Supplementary Information

Below is the link to the electronic supplementary material.

ESM 1 (DOCX 2.67 MB)

## Data Availability

The data supporting the findings of this study are not publicly available due to confidentiality restrictions.

## Abbreviations

*BMI*: Body mass index
*ITB*: Iliotibial band
*KL*: Kellgren and Lawrence system
*KPM*: Knee Pain Map
*MCL*: Medial collateral ligament
*LCL*: Lateral collateral ligament
*NRS*: Numeric rating scale
*OA*: Osteoarthritis
*OMERACT*: Outcome Measures in Rheumatology
*PD*: Power Doppler
*SMT*: Semimembranosus tendon
*US*: Ultrasound
*VAS*: Visual analogue scale
