# Supplementary material for: The relationship between real-time ultrasound-detected extra-articular soft tissue pathologies and knee pain in osteoarthritis: a cross-sectional study
Source: Clin Rheumatol. 2026 Mar 6;45(5):2837–44. doi: 10.1007/s10067-026-08017-x (PMC13068718; doi:10.1007/s10067-026-08017-x)
Supplement: Supplementary file 1 — (DOCX 2.67 MB) [file 10067_2026_8017_MOESM1_ESM.docx]

**Knee pain map**

The Knee Pain Map is a validated tool used to assess knee pain location, allowing patients to point to the specific site of their pain. Such pain maps have been studied in the development of validated methods for assessing pain distribution [10]. Pain patterns on the Knee Pain Map were described as: localised (with eight possible locations of superior-medial, inferior-medial, medial joint line, superior-lateral, inferior-lateral, lateral joint line, patellar or back of knee); regional (with four possible locations of medial, lateral, patellar, or back of the knee); or diffuse/unable to identify pain as localised or regional in nature. These locations were marked on an artist’s drawing of the knee (Supplementary figure 1). If only one or two fingers pointed to a specific location the pain pattern was “localised”. If all fingers were used the pain was or a whole hand covered a broader region the pain was “regional”. If they were unable to identify a location or region or the pain was described as “all over” the pain was described as “diffuse”. Participants could report pain in more than one region. The Knee Pain Map has a high interrater reliability for identifying localised and regional pain [10].

# **SUPPLEMENTARY FIGURE 1 Knee Pain Map**


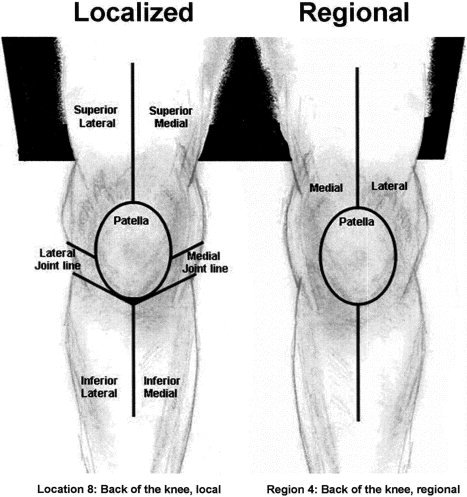


Taken from Thompson et al [10]

**SUPPLEMENTARY FIGURE 2 – Knee pain map with annotated structural landmarks**

**Quadriceps tendon**


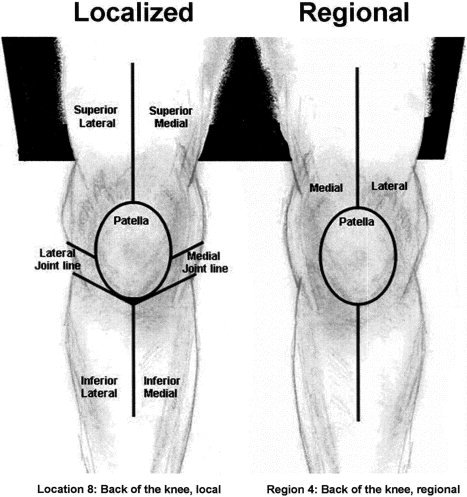


**Patellar tendon**

**Infrapatellar bursitis**

**Anserine bursitis**

**Lateral collateral ligament**

**Semi membranosus tendon (back of knee)**

**Iliotibial band insertion**

**Iliotibial band bursitis**

**Medial collateral ligament**

**Baker’s cyst (back of knee)**

**Quadriceps tendon**

**SUPPLEMENTARY FIGURE 3 – Ultrasound probe positioning**


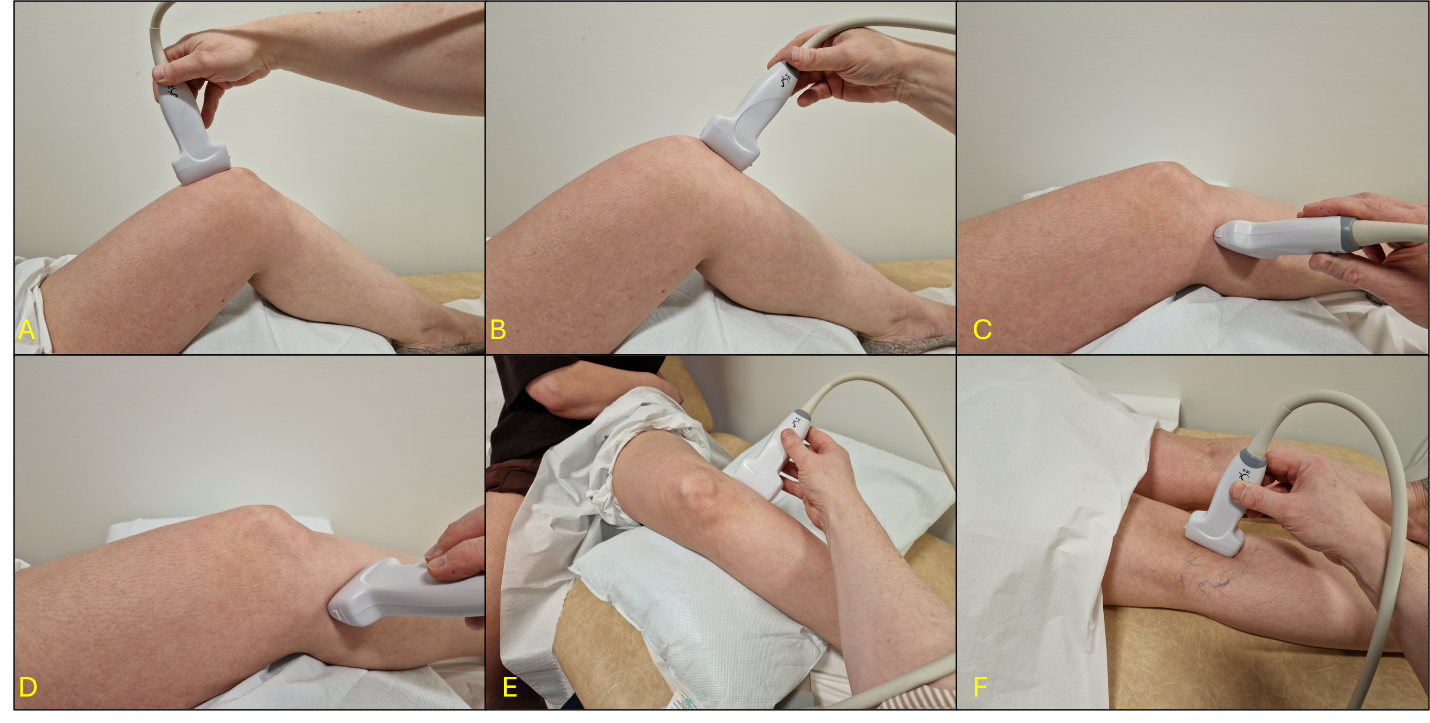


**Probe positions used for US assessment.** Panels A and B illustrate anterior probe positions for suprapatellar and infrapatellar views, respectively. Panel C shows the medial probe position at the distal insertion of the medial collateral ligament (MCL), while Panel D shows the medial probe position for assessment of the pes anserine bursa. Panel E shows the lateral assessment, and Panel F shows the posterior assessment.

**SUPPLEMENTARY FIGURE 4 – Quadriceps enthesophyte**

**
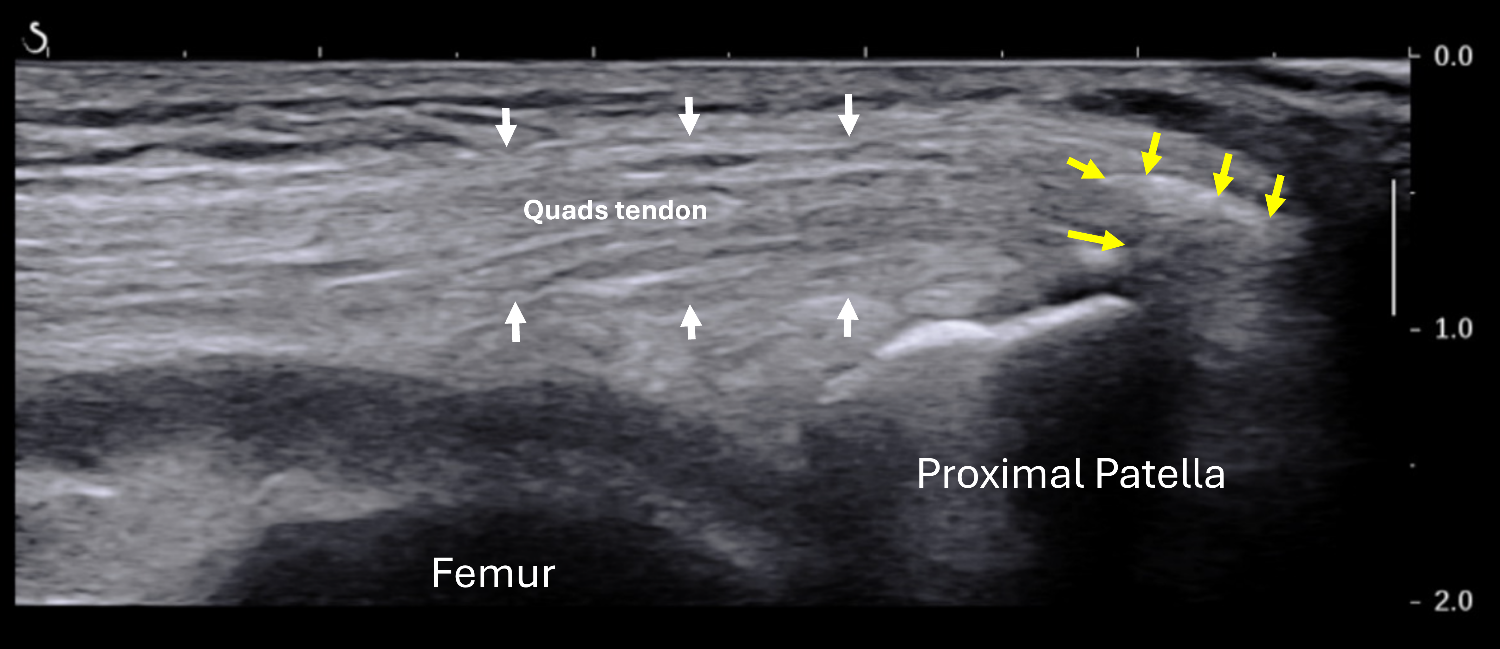
**

Longitudinal ultrasound section through the midline of the proximal knee. The edges of the quadriceps tendon are noted with white arrows. A large enthesophyte is noted by the yellow arrows.

**SUPPLEMENTARY FIGURE 5: Locations of sono-palpation**


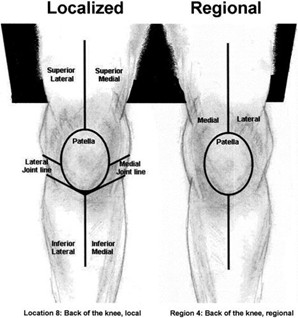


Circles with white centre represent posterior knee tenderness locations

**SUPPLEMENTARY TABLE 1: US CHARACTERISTICS BY REGION WITH GRADING**

| **Quadrant** | **Structure** | **US characteristic (scale)*** | | | | | | | | |
| --- | --- | --- | --- | --- | --- | --- | --- | --- | --- | --- |
| Anterior |  | **Presence (0-1)** | **Thickening/enlarged (0-1)** | **Grade (0-3)** | **Hypoechogeneity (0-1)** | **PD grade (0-3)** | **Insert. Erosions (0-1)** | **Enthesophytes (0-1)** | **Tear (0-1)** | **Sono-palpation (0-1)** |
|  | Quadriceps tendon | NA |  |  |  |  |  |  |  |  |
|  | Patellar tendon (proximal) | NA |  |  |  |  |  |  |  |  |
|  | Patellar tendon  (distal) | NA |  |  |  |  |  |  |  |  |
|  | Deep infrapatellar bursitis |  | NA |  | NA |  | NA | NA | NA |  |
|  | Superficial infrapatellar bursitis |  | NA |  | NA |  | NA | NA | NA |  |
| Medial | MCL origin | NA |  |  |  |  |  |  |  |  |
|  | MCL insertion | NA |  |  |  |  |  |  |  |  |
|  | Anserine bursitis |  | NA |  | NA |  | NA | NA | NA |  |
| Lateral | LCL origin | NA |  |  |  |  |  |  |  |  |
|  | LCL insertion | NA |  |  |  |  |  |  |  |  |
|  | ITB insertion | NA |  |  |  |  |  |  |  |  |
|  | ITB bursitis |  | NA |  | NA |  | NA | NA | NA |  |
| Posterior | SMT insertion | NA |  |  |  |  |  |  |  |  |
|  | Baker’s cyst |  |  |  | NA | NA | NA | NA | NA |  |

*For semi quantitative assessment scoring, grade meaning: 0: Absent; 1: Minimal; 2: Moderate; 3: Severe. For binary scoring, grade meaning: 0: Absent; 1: Present

**SUPPLEMENTARY TABLE 2 US pathology frequencies by quadrant location**

| **Quadrant/structure** | |  |  |  |  |  |  |  |  |  |  |  |  |  |  |  |  |
| --- | --- | --- | --- | --- | --- | --- | --- | --- | --- | --- | --- | --- | --- | --- | --- | --- | --- |
|  | ***Presence*** | | | ***Thickening/enlarged*** | | ***Hypoechogenicity*** | | ***PD grade >0*** | | ***Insertion erosions*** | | ***Enthesophytes*** | | ***Ligament/tendon Tear*** | | ***Sono-palpation*** | |
|  | N | | *%* | N | *%* | N | *%* | N | *%* | N | *%* | N | *%* | N | *%* | N | *%* |
| **Anterior** | | | | | | | | | | | | | | | | | |
| Quadriceps tendon |  | |  |  |  |  |  |  |  |  |  |  |  |  |  |  |  |
| *Present* | N/A | | N/A | 9 | 16.67 | 9 | 16.67 | 1 | 1.85 | 1 | 1.85 | 28 | 51.85 | 2 | 3.70 | 11 | 20.37 |
| *Absent* | N/A | | N/A | 45 | 83.33 | 45 | 83.33 | 53 | 98.15 | 53 | 98.15 | 26 | 48.15 | 52 | 96.40 | 43 | 79.63 |
| Proximal/distal patellar tendon |  | |  |  |  |  |  |  |  |  |  |  |  |  |  |  |  |
| *Present* | N/A | | N/A | 5 | 9.26 | 9 | 16.67 | 2 | 3.70 | 0 | 0 | 9 | 16.67 | 0 | 0 | 3 | 5.56 |
| *Absent* | N/A | | N/A | 49 | 90.74 | 45 | 83.33 | 52 | 96.30 | 54 | 100 | 45 | 83.33 | 54 | 100 | 51 | 94.44 |
| Deep/superficial infrapatellar bursitis |  | |  |  |  |  |  |  |  |  |  |  |  |  |  |  |  |
| *Present* | 9 | | 16.67 | N/A | N/A | N/A | N/A | 0 | 0 | N/A | N/A | N/A | N/A | N/A | N/A | 1 | 1.85 |
| *Absent* | 45 | | 83.33 | N/A | N/A | N/A | N/A | 54 | 100 | N/A | N/A | N/A | N/A | N/A | N/A | 53 | 98.15 |
| **Medial** | | | | | | | | | | | | | | | | | |
| Anserine bursitis |  | |  |  |  |  |  |  |  |  |  |  |  |  |  |  |  |
| *Present* | 2 | | 3.70 | N/A | N/A | N/A | N/A | 0 | 0 | N/A | N/A | N/A | N/A | N/A | N/A | 0 | 0 |
| *Absent* | 52 | | 96.30 | N/A | N/A | N/A | N/A | 54 | 100 | N/A | N/A | N/A | N/A | N/A | N/A | 54 | 100 |
| MCL origin/insertion |  | |  |  |  |  |  |  |  |  |  |  |  |  |  |  |  |
| *Present* | N/A | | N/A | 10 | 18.52 | 13 | 24.07 | 6 | 11.11 | 4 | 7.41 | 11 | 20.37 | 0 | 0 | 21 | 38.89 |
| *Absent* | N/A | | N/A | 44 | 81.48 | 41 | 75.93 | 47 | 87.04 | 50 | 92.59 | 43 | 79.63 | 54 | 100 | 33 | 61.11 |
| **Lateral** | | | | | | | | | | | | | | | | | |
| LCL origin/insertion |  | |  |  |  |  |  |  |  |  |  |  |  |  |  |  |  |
| *Present* | N/A | | N/A | 18 | 33.33 | 21 | 38.89 | 3 | 5.56 | 3 | 5.56 | 10 | 18.52 | 2 | 3.70 | 20 | 37.04 |
| *Absent* | N/A | | N/A | 36 | 66.67 | 33 | 61.11 | 51 | 94.44 | 51 | 94.44 | 44 | 81.48 | 52 | 96.30 | 34 | 62.96 |
| ITB insertion |  | |  |  |  |  |  |  |  |  |  |  |  |  |  |  |  |
| *Present* | N/A | | N/A | 3 | 5.56 | 6 | 11.11 | 1 | 1.85 | 1 | 1.85 | N/A | N/A | 0 | 0 | 5 | 9.26 |
| *Absent* | N/A | | N/A | 51 | 94.44 | 48 | 88.89 | 53 | 98.15 | 53 | 98.15 | N/A | N/A | 54 | 100 | 49 | 90.74 |
| ITB bursitis |  | |  |  |  |  |  |  |  |  |  |  |  |  |  |  |  |
| *Present* | 1 | | 1.85 | 2 | 3.70 | N/A | N/A | 1 | 1.85 | N/A | N/A | N/A | N/A | N/A | N/A | N/A | N/A |
| *Absent* | 53 | | 98.15 | 52 | 96.30 | N/A | N/A | 53 | 98.15 | N/A | N/A | N/A | N/A | N/A | N/A | N/A | N/A |
| **Posterior** | | | | | | | | | | | | | | | | | |
| Baker's cyst |  | |  |  |  |  |  |  |  |  |  |  |  |  |  |  |  |
| *Present* | 20 | | 37.04 | 25 | 46.30 | N/A | N/A | N/A | N/A | N/A | N/A | N/A | N/A | N/A | N/A | 6 | 11.11 |
| *Absent* | 34 | | 53.70 | 29 | 53.70 | N/A | N/A | N/A | N/A | N/A | N/A | N/A | N/A | N/A | N/A | 47 | 87.04 |
| SMT insertion |  | |  |  |  |  |  |  |  |  |  |  |  |  |  |  |  |
| *Present* | N/A | | N/A | 17 | 31.45 | 29 | 53.70 | 2 | 3.70 | 11 | 20.37 | 18 | 33.33 | 0 | 0 | 36 | 66.67 |
| *Absent* | N/A | | N/A | 36 | 66.67 | 24 | 44.44 | 51 | 94.44 | 42 | 77.76 | 34 | 62.96 | 50 | 100 | 18 | 33.33 |

**SUPPLEMENTARY TABLE 3: All pathology frequences in each knee region**

| Region | Pathology | Frequency of pathology*† |
| --- | --- | --- |
| Anterior | Semi-membranosus tendon hypoechogeneity | 55.6% (5/9) |
|  | Semi-membranosus tendon sono-palpation | 50% (5/10) |
|  | Medial collateral ligament sono-palpation | 50% (5/10) |
|  | Quadriceps tendon enthesophytes | 50% (5/10) |
|  | Semi-membranosus tendon enthesophytes | 44.4% (4/9) |
|  | Medial collateral ligament hypoechogeneity | 40% (4/10) |
|  | Lateral collateral ligament hypoechogeneity | 40% (4/10) |
|  | Lateral collateral ligament thickening | 40% (4/10) |
|  | Lateral collateral ligament enthesophytes | 40% (4/10) |
|  | Semi-membranosus tendon grade | 33.3% (3/9) |
|  | Patellar tendon enthesophytes | 30% (3/10) |
|  | Patellar tendon thickening | 30% (3/10) |
|  | Baker's cyst thickening | 30% (3/10) |
|  | Medial collateral ligament thickening | 30% (3/10) |
|  | Baker's cyst sono-palpation | 22.2% (2/9) |
|  | Semi-membranosus tendon insertion erosion | 22.2% (2/9) |
|  | Semi-membranosus tendon thickening | 22.2% (2/9) |
|  | Patellar tendon sono-palpation | 20% (2/10) |
|  | Baker's cyst presence | 20% (2/10) |
|  | Lateral collateral ligament sono-palpation | 20% (2/10) |
|  | Medial collateral ligament enthesophytes | 20% (2/10) |
|  | Medial collateral ligament erosion | 20% (2/10) |
|  | Proximal patellar tendon thickening | 20% (2/10) |
|  | Quadriceps tendon hypoechogeneity | 20% (2/10) |
|  | Medial collateral ligament power Doppler grade | 11.1% (1/9) |
|  | Anserine bursitis grade | 10% (1/10) |
|  | Anserine bursitis presence | 10% (1/10) |
|  | Anserine bursitis sono-palpation | 10% (1/10) |
|  | Infrapatellar bursitis presence | 10% (1/10) |
|  | Patellar tendon hypoechogeneity | 10% (1/10) |
|  | Lateral collateral ligament erosion | 10% (1/10) |
|  | Lateral collateral ligament power Doppler grade | 10% (1/10) |
|  | Lateral collateral ligament tear | 10% (1/10) |
|  | Quadriceps tendon erosion | 10% (1/10) |
|  | Quadriceps tendon sono-palpation | 10% (1/10) |
|  | Anserine bursitis power Doppler grade | 0% (0/10) |
|  | Infrapatellar bursitis power Doppler grade | 0% (0/10) |
|  | Infrapatellar bursitis sono-palpation | 0% (0/10) |
|  | Patellar tendon power Doppler grade | 0% (0/10) |
|  | Patellar tendon tear | 0% (0/10) |
|  | Iliotibial band bursitis presence | 0% (0/10) |
|  | Iliotibial band bursitis sono-palpation | 0% (0/10) |
|  | Iliotibial band bursitis thickening | 0% (0/10) |
|  | Iliotibial band bursitis power Doppler grade | 0% (0/10) |
|  | Iliotibial band insertion power Doppler grade | 0% (0/10) |
|  | Medial collateral ligament tear | 0% (0/10) |
|  | Patellar tendon erosion | 0% (0/10) |
|  | Quadriceps tendon power Doppler grade | 0% (0/10) |
|  | Quadriceps tendon tear | 0% (0/10) |
|  | Semi-membranosus tendon power Doppler grade | 0% (0/9) |
|  | Semi-membranosus tendon tear | 0% (0/9) |
| Medial | Semi-membranosus tendon sono-palpation | 70.3% (26/37) |
|  | Quadriceps tendon enthesophytes | 56.8% (21/37) |
|  | Semi-membranosus tendon hypoechogeneity | 54.1% (20/37) |
|  | Medial collateral ligament sono-palpation | 43.2% (16/37) |
|  | Semi-membranosus tendon enthesophytes | 41.7% (15/36) |
|  | Semi-membranosus tendon grade | 40.5% (15/37) |
|  | Lateral collateral ligament sono-palpation | 37.8% (14/37) |
|  | Baker's cyst thickening | 35.1% (13/37) |
|  | Lateral collateral ligament hypoechogeneity | 35.1% (13/37) |
|  | Semi-membranosus tendon thickening | 32.4% (12/37) |
|  | Baker's cyst presence | 27% (10/37) |
|  | Lateral collateral ligament thickening | 27% (10/37) |
|  | Quadriceps tendon sono-palpation | 24.3% (9/37) |
|  | Quadriceps tendon hypoechogeneity | 21.6% (8/37) |
|  | Semi-membranosus tendon insertion erosion | 21.6% (8/37) |
|  | Medial collateral ligament hypoechogeneity | 18.9% (7/37) |
|  | Infrapatellar bursitis presence | 16.2% (6/37) |
|  | Patellar tendon enthesophytes | 16.2% (6/37) |
|  | Patellar tendon hypoechogeneity | 16.2% (6/37) |
|  | Lateral collateral ligament enthesophytes | 16.2% (6/37) |
|  | Medial collateral ligament enthesophytes | 16.2% (6/37) |
|  | Medial collateral ligament power Doppler grade | 13.5% (5/37) |
|  | Medial collateral ligament thickening | 13.5% (5/37) |
|  | Anserine bursitis sono-palpation | 8.1% (3/37) |
|  | Lateral collateral ligament erosion | 5.4% (2/37) |
|  | Lateral collateral ligament power Doppler grade | 5.4% (2/37) |
|  | Medial collateral ligament erosion | 5.4% (2/37) |
|  | Baker's cyst sono-palpation | 2.8% (1/36) |
|  | Anserine bursitis presence | 2.7% (1/37) |
|  | Patellar tendon power Doppler grade | 2.7% (1/37) |
|  | Patellar tendon sono-palpation | 2.7% (1/37) |
|  | Patellar tendon thickening | 2.7% (1/37) |
|  | Iliotibial band bursitis sono-palpation | 2.7% (1/37) |
|  | Iliotibial band bursitis thickening | 2.7% (1/37) |
|  | Iliotibial band bursitis power Doppler grade | 2.7% (1/37) |
|  | Iliotibial band insertion power Doppler grade | 2.7% (1/37) |
|  | Proximal patellar tendon thickening | 2.7% (1/37) |
|  | Quadriceps tendon erosion | 2.7% (1/37) |
|  | Quadriceps tendon power Doppler grade | 2.7% (1/37) |
|  | Semi-membranosus tendon power Doppler grade | 2.7% (1/37) |
|  | Anserine bursitis grade | 0% (0/36) |
|  | Infrapatellar bursitis power Doppler grade | 0% (0/37) |
|  | Infrapatellar bursitis sono-palpation | 0% (0/37) |
|  | Patellar tendon erosion | 0% (0/37) |
|  | Patellar tendon tear | 0% (0/37) |
|  | Iliotibial band bursitis presence | 0% (0/37) |
|  | Lateral collateral ligament tear | 0% (0/37) |
|  | Anserine bursitis power Doppler grade | 0% (0/37) |
|  | Medial collateral ligament tear | 0% (0/37) |
|  | Quadriceps tendon tear | 0% (0/37) |
|  | Semi-membranosus tendon tear | 0% (0/34) |
| Lateral | Semi-membranosus tendon sono-palpation | 82.4% (14/17) |
|  | Semi-membranosus tendon hypoechogeneity | 81.2% (13/16) |
|  | Baker's cyst thickening | 52.9% (9/17) |
|  | Lateral collateral ligament sono-palpation | 52.9% (9/17) |
|  | Semi-membranosus tendon grade | 50% (8/16) |
|  | Medial collateral ligament sono-palpation | 47.1% (8/17) |
|  | Semi-membranosus tendon thickening | 43.8% (7/16) |
|  | Baker's cyst presence | 41.2% (7/17) |
|  | Patellar tendon enthesophytes | 29.4% (5/17) |
|  | Lateral collateral ligament hypoechogeneity | 29.4% (5/17) |
|  | Quadriceps tendon enthesophytes | 29.4% (5/17) |
|  | Semi-membranosus tendon enthesophytes | 25% (4/16) |
|  | Patellar tendon hypoechogeneity | 23.5% (4/17) |
|  | Baker's cyst sono-palpation | 23.5% (4/17) |
|  | Lateral collateral ligament thickening | 23.5% (4/17) |
|  | Medial collateral ligament hypoechogeneity | 23.5% (4/17) |
|  | Medial collateral ligament thickening | 23.5% (4/17) |
|  | Quadriceps tendon sono-palpation | 23.5% (4/17) |
|  | Semi-membranosus tendon insertion erosion | 18.8% (3/16) |
|  | Infrapatellar bursitis presence | 17.6% (3/17) |
|  | Medial collateral ligament enthesophytes | 17.6% (3/17) |
|  | Anserine bursitis sono-palpation | 11.8% (2/17) |
|  | Patellar tendon sono-palpation | 11.8% (2/17) |
|  | Patellar tendon thickening | 11.8% (2/17) |
|  | Lateral collateral ligament enthesophytes | 11.8% (2/17) |
|  | Medial collateral ligament erosion | 11.8% (2/17) |
|  | Quadriceps tendon hypoechogeneity | 11.8% (2/17) |
|  | Semi-membranosus tendon power Doppler grade | 6.2% (1/16) |
|  | Anserine bursitis presence | 5.9% (1/17) |
|  | Patellar tendon power Doppler grade | 5.9% (1/17) |
|  | Medial collateral ligament power Doppler grade | 5.9% (1/17) |
|  | Proximal patellar tendon thickening | 5.9% (1/17) |
|  | Quadriceps tendon power Doppler grade | 5.9% (1/17) |
|  | Semi-membranosus tendon tear | 0% (0/15) |
|  | Anserine bursitis grade | 0% (0/16) |
|  | Anserine bursitis power Doppler grade | 0% (0/17) |
|  | Infrapatellar bursitis power Doppler grade | 0% (0/17) |
|  | Infrapatellar bursitis sono-palpation | 0% (0/17) |
|  | Patellar tendon erosion | 0% (0/17) |
|  | Patellar tendon tear | 0% (0/17) |
|  | Iliotibial band bursitis presence | 0% (0/17) |
|  | Iliotibial band bursitis sono-palpation | 0% (0/17) |
|  | Iliotibial band bursitis thickening | 0% (0/17) |
|  | Iliotibial band bursitis power Doppler grade | 0% (0/17) |
|  | Iliotibial band insertion power Doppler grade | 0% (0/17) |
|  | Lateral collateral ligament power Doppler grade | 0% (0/17) |
|  | Lateral collateral ligament tear | 0% (0/17) |
|  | Lateral collateral ligament erosion | 0% (0/17) |
|  | Medial collateral ligament tear | 0% (0/17) |
|  | Quadriceps tendon erosion | 0% (0/17) |
|  | Quadriceps tendon tear | 0% (0/17) |
| Posterior | Baker's cyst thickening | 80% (4/5) |
|  | Semi-membranosus tendon sono-palpation | 80% (4/5) |
|  | Quadriceps tendon enthesophytes | 60% (3/5) |
|  | Baker's cyst presence | 60% (3/5) |
|  | Medial collateral ligament hypoechogeneity | 40% (2/5) |
|  | Semi-membranosus tendon enthesophytes | 40% (2/5) |
|  | Semi-membranosus tendon grade | 40% (2/5) |
|  | Semi-membranosus tendon hypoechogeneity | 40% (2/5) |
|  | Baker's cyst sono-palpation | 20% (1/5) |
|  | Infrapatellar bursitis presence | 20% (1/5) |
|  | Patellar tendon enthesophytes | 20% (2/5) |
|  | Patellar tendon hypoechogeneity | 20% (2/5) |
|  | Patellar tendon thickening | 20% (2/5) |
|  | Medial collateral ligament power Doppler grade | 20% (2/5) |
|  | Medial collateral ligament sono-palpation | 20% (2/5) |
|  | Medial collateral ligament thickening | 20% (2/5) |
|  | Proximal patellar tendon thickening | 20% (2/5) |
|  | Quadriceps tendon hypoechogeneity | 20% (2/5) |
|  | Semi-membranosus tendon thickening | 20% (2/5) |
|  | Anserine bursitis grade | 0% (0/5) |
|  | Anserine bursitis presence | 0% (0/5) |
|  | Anserine bursitis sono-palpation | 0% (0/5) |
|  | Infrapatellar bursitis power Doppler grade | 0% (0/5) |
|  | Infrapatellar bursitis sono-palpation | 0% (0/5) |
|  | Patellar tendon erosion | 0% (0/5) |
|  | Patellar tendon power Doppler grade | 0% (0/5) |
|  | Patellar tendon tear | 0% (0/5) |
|  | Patellar tendon sono-palpation | 0% (0/5) |
|  | Iliotibial band bursitis presence | 0% (0/5) |
|  | Iliotibial band bursitis sono-palpation | 0% (0/5) |
|  | Iliotibial band bursitis thickening | 0% (0/5) |
|  | Iliotibial band bursitis power Doppler grade | 0% (0/5) |
|  | Iliotibial band insertion power Doppler grade | 0% (0/5) |
|  | Lateral collateral ligament enthesophytes | 0% (0/5) |
|  | Lateral collateral ligament erosion | 0% (0/5) |
|  | Lateral collateral ligament hypoechogeneity | 0% (0/5) |
|  | Lateral collateral ligament power Doppler grade | 0% (0/5) |
|  | Lateral collateral ligament tear | 0% (0/5) |
|  | Lateral collateral ligament sono-palpation | 0% (0/5) |
|  | Lateral collateral ligament thickening | 0% (0/5) |
|  | Anserine bursitis power Doppler grade | 0% (0/5) |
|  | Medial collateral ligament enthesophytes | 0% (0/5) |
|  | Medial collateral ligament erosion | 0% (0/5) |
|  | Medial collateral ligament tear | 0% (0/5) |
|  | Quadriceps tendon erosion | 0% (0/5) |
|  | Quadriceps tendon power Doppler grade | 0% (0/5) |
|  | Quadriceps tendon tear | 0% (0/5) |
|  | Quadriceps tendon sono-palpation | 0% (0/5) |
|  | Semi-membranosus tendon insertion erosion | 0% (0/5) |
|  | Semi-membranosus tendon power Doppler grade | 0% (0/5) |
|  | Semi-membranosus tendon tear | 0% (0/5) |
| Diffuse | Quadriceps tendon enthesophytes | 60% (3/5) |
|  | Medial collateral ligament enthesophytes | 60% (3/5) |
|  | Baker's cyst thickening | 60% (3/5) |
|  | Baker's cyst presence | 40% (2/5) |
|  | Lateral collateral ligament hypoechogeneity | 40% (2/5) |
|  | Lateral collateral ligament thickening | 40% (2/5) |
|  | Medial collateral ligament hypoechogeneity | 40% (2/5) |
|  | Medial collateral ligament thickening | 40% (2/5) |
|  | Semi-membranosus tendon grade | 40% (2/5) |
|  | Semi-membranosus tendon hypoechogeneity | 40% (2/5) |
|  | Semi-membranosus tendon sono-palpation | 40% (2/5) |
|  | Semi-membranosus tendon thickening | 40% (2/5) |
|  | Iliotibial band bursitis presence | 20% (1/5) |
|  | Iliotibial band bursitis thickening | 20% (1/5) |
|  | Lateral collateral ligament enthesophytes | 20% (1/5) |
|  | Lateral collateral ligament sono-palpation | 20% (1/5) |
|  | Medial collateral ligament sono-palpation | 20% (1/5) |
|  | Quadriceps tendon sono-palpation | 20% (1/5) |
|  | Semi-membranosus tendon enthesophytes | 20% (1/5) |
|  | Anserine bursitis grade | 0% (0/5) |
|  | Anserine bursitis power Doppler grade | 0% (0/5) |
|  | Anserine bursitis presence | 0% (0/5) |
|  | Anserine bursitis sono-palpation | 0% (0/5) |
|  | Infrapatellar bursitis power Doppler grade | 0% (0/5) |
|  | Infrapatellar bursitis presence | 0% (0/5) |
|  | Infrapatellar bursitis sono-palpation | 0% (0/5) |
|  | Patellar tendon enthesophytes | 0% (0/5) |
|  | Patellar tendon erosion | 0% (0/5) |
|  | Patellar tendon hypoechogeneity | 0% (0/5) |
|  | Patellar tendon power Doppler grade | 0% (0/5) |
|  | Patellar tendon tear | 0% (0/5) |
|  | Patellar tendon sono-palpation | 0% (0/5) |
|  | Patellar tendon thickening | 0% (0/5) |
|  | Baker's cyst sono-palpation | 0% (0/5) |
|  | Iliotibial band bursitis sono-palpation | 0% (0/5) |
|  | Iliotibial band bursitis power Doppler grade | 0% (0/5) |
|  | Iliotibial band insertion power Doppler grade | 0% (0/5) |
|  | Lateral collateral ligament erosion | 0% (0/5) |
|  | Lateral collateral ligament power Doppler grade | 0% (0/5) |
|  | Lateral collateral ligament tear | 0% (0/5) |
|  | Medial collateral ligament erosion | 0% (0/5) |
|  | Medial collateral ligament power Doppler grade | 0% (0/5) |
|  | Medial collateral ligament tear | 0% (0/5) |
|  | Proximal patellar tendon thickening | 0% (0/5) |
|  | Quadriceps tendon erosion | 0% (0/5) |
|  | Quadriceps tendon hypoechogeneity | 0% (0/5) |
|  | Quadriceps tendon power Doppler grade | 0% (0/5) |
|  | Quadriceps tendon tear | 0% (0/5) |
|  | Semi-membranosus tendon insertion erosion | 0% (0/5) |
|  | Semi-membranosus tendon power Doppler grade | 0% (0/5) |
|  | Semi-membranosus tendon tear | 0% (0/5) |

*Out of total number of participants with pain in specified region

†Denominator may reduce due to missing ultrasound variable data
